# Addressing disparities in the health of persons with HIV attributable to unstable housing in the United States: The role of the Ryan White HIV/AIDS Program

**DOI:** 10.1371/journal.pmed.1003057

**Published:** 2020-03-02

**Authors:** Amy Griffin, Antigone Dempsey, Wendy Cousino, Latham Avery, Harold Phillips, Emeka Egwim, Laura Cheever

**Affiliations:** US Department of Health and Human Services, Health Resources and Services Administration, HIV/AIDS Bureau, Division of Policy and Data, Rockville, Maryland, United States of America

## Abstract

Amy Griffin and co-authors discuss unstable housing and the response to HIV/AIDS in the United States.

**Summary points**

- There are an estimated 1.1 million people aged 13 and older who have HIV in the United States, including an estimated 162,500 (14%) people whose infections had not been diagnosed. In 2017, 38,281 people received an HIV diagnosis in the US and dependent areas.
- Unstably housed people with HIV are a key population for ending the HIV epidemic in the US, because of their difficulty accessing HIV care, high rates of mental health disorders, substance use, and economic and food insecurity, which can lead to poor health outcomes and low viral suppression.
- The Health Resources and Services Administration’s Ryan White HIV/AIDS Program (HRSA RWHAP) serves more than half of all people with diagnosed HIV in the United States, including over 25,000 unstably housed people. From 2010 through 2017, the percentage of RWHAP clients with stable housing increased from 82% to 87%.
- However, only 71% of unstably housed RWHAP clients were virally suppressed, compared with 86% of all RWHAP clients. This is lower than rates of viral suppression in other key population groups like youth (74%) and transgender people (81%).
- HRSA and the RWHAP promote increased access to housing services for people with HIV through (a) innovative federal partnerships with the Department of Housing and Urban Development, (b) policy guidance for HRSA grant recipients, and (c) developing and implementing innovative models and interventions.

## Introduction

Housing has emerged as a key element of healthcare for improving health outcomes for people with HIV. Research indicates that unstable housing and the degree of instability (including people experiencing homelessness) profoundly impact the ability of people with HIV to access HIV medical care to achieve and sustain viral suppression [1–3].

At the end of 2016, an estimated 1.1 million people aged 13 and older had HIV in the United States, including an estimated 162,500 (14%) people whose infections had not been diagnosed [4]. In 2017, 38,281 people received an HIV diagnosis in the US and dependent areas [5]. Although the number of newly diagnosed individuals continues to decline in the US, some Americans, including African-Americans, men who have sex with men (MSM), and those identified as unstably housed continue to be disproportionately impacted by HIV [6]. As the nation moves forward with a structured approach toward Ending the Epidemic, it will be important for communities to assess and address the needs of those Americans most likely to be diagnosed with HIV and least likely to achieve viral suppression in our healthcare systems.

Nationally, there are many contributing factors that impact the homelessness rates and housing instability in the US. Housing instability rarely happens without the additional challenges of mental health disorders, substance use, and economic and food insecurity, which can further complicate healthcare needs [7]. In 2018, the US Department of Housing and Urban Development’s (HUD) national data showed that on a single night in January, in all states, territories, Puerto Rico, and the District of Columbia, 111,122 persons with severe mental illness, 86,647 with chronic substance use, and 10,064 with HIV were living in emergency shelters, transitional facilities, or unsheltered [8].

Of the 534,903 people served by the Health Resources and Services Administration’s (HRSA) Ryan White HIV/AIDS Program (RWHAP) in 2017, 7.8% reported being temporarily housed, and 5.1% reported unstable housing. Those who reported temporary or unstable housing, regardless of sociodemographic factors (e.g., by gender, risk factor, race and ethnicity, age, poverty level, and geographic location) were less likely to be virally suppressed, demonstrating a significant health disparity caused by unstable and temporary housing [9]. To address these challenges, HRSA has implemented a number of strategies including aligning federal partnerships and leveraging resources, addressing policies to increase use of housing services, and funding initiatives to better understand how to address housing needs for people with HIV to address the challenges in retention in care and viral suppression faced by people with HIV who are temporarily or unstably housed.

### The RWHAP and housing status data

HRSA’s RWHAP funds and coordinates a comprehensive system of over 2,000 provider sites. These provider sites include states, cities, universities, and clinics funded by HRSA to deliver HIV care (e.g., medical services, substance use disorder treatment, mental health), medications (both HIV antiretrovirals and for HIV-related conditions), and support services (e.g., case management, housing, transportation) for low-income, uninsured, or underserved people with HIV. In 2017, the RWHAP served 534,903 clients, representing more than 50% of people with diagnosed HIV in the US [5]. Client-level data are collected by funded provider sites [9] and include sociodemographics (gender, risk factor, age, poverty level, geographic location, race, and ethnicity), services provided, and HIV health outcomes (retention in care and viral suppression status). Housing status data are also collected and organized into 3 categories: stably housed (permanent housing), temporarily housed (transitional housing, temporary housing with family, or institutional), or unstably housed (homeless or living in an emergency shelter or place not designed for human accommodation such as a vehicle or abandoned building).

### Differences in housing status and client characteristics

Of the RWHAP clients who reported their housing status from 2010 through 2017, the percentages of those stably housed increased from 82.0% to 87.1%. Those temporarily housed decreased from 14.2% to 7.8%, and those unstably housed increased from 3.8% to 5.1% (Table 1).

**Table 1:** RWHAP Services Report, clients by housing status, 2010–2017—US and 3 territories.

RWHAP Client Housing Status (%)			
Year	Stable	Temporary	Unstable
2010	82.0	14.2	3.8
2011	82.4	13.7	3.8
2012	83.1	12.9	4.1
2013	83.3	12.4	4.3
2014	83.5	11.8	4.7
2015	85.2	9.8	5.0
2016	86.1	8.8	5.2
2017	87.1	7.8	5.1
Net Change	5.1	−5.4	1.3

*Source*: *HRSA*. *RWHAP Services Report (RSR) 2017*. Does not include AIDS Drug Assistance Program data [9]. HSRA, Health Resources and Services Administration; RWHAP, Ryan White HIV/AIDS Program.

Through the dedicated efforts of care teams at HRSA RWHAP provider sites, housing status overall has improved by decreasing the number of people with HIV who have temporary housing. However, the proportion who are unstably housed has continued to increase, highlighting differences between the temporarily housed and unstably housed populations. A limitation of the RWHAP data is its current inability to track clients across years. An important question that needs to be addressed is, “How many clients who report living in an unstable housing situation have been unstably housed for multiple years?” A recent HRSA RWHAP nationally funded initiative, Building a Medical Home for Multiply Diagnosed HIV-Positive Homeless Populations, reported that the mean number of years of homelessness reported by participants was over 6 years. Additionally, the ability to achieve stable housing was associated with lower levels of substance use, recent incarceration, and unmet needs, as well as higher levels of self-efficacy and social support [10].

**Fig 1:**
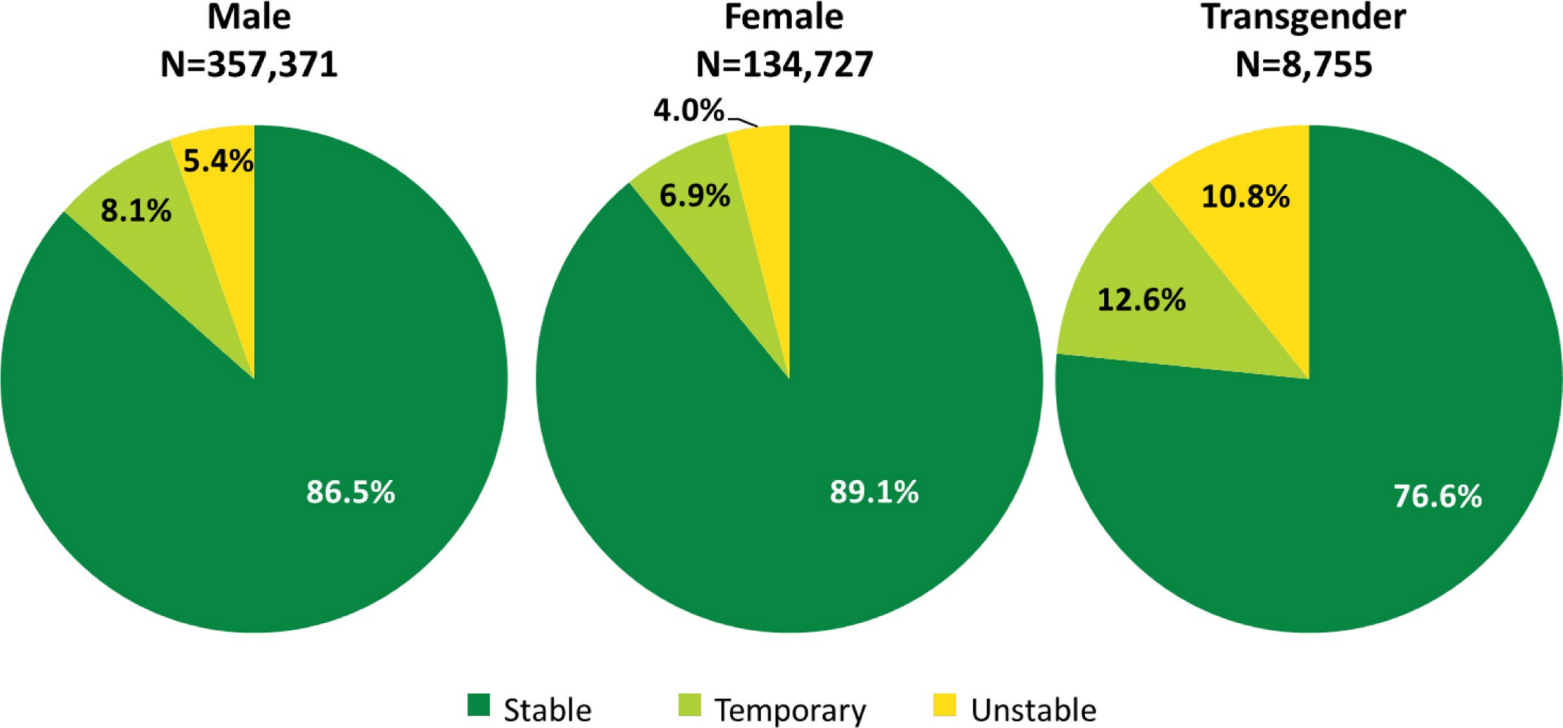
RWHAP clients by gender and housing Status, 2017—US and 3 territories. *Source*: *HRSA*. *RWHAP Services Report (RSR) 2017*. Does not include AIDS Drug Assistance Program Data. [9] HSRA, Health Resources and Services Administration; RWHAP, Ryan White HIV/AIDS Program.

Disparities in housing status were also reported by gender. Among transgender individuals, 12.6% had temporary housing, and 10.8% had unstable housing compared with men (8.1% and 5.4%, respectively), and women (6.9% and 4%, respectively) (Fig 1) [9]. Transgender people face even greater barriers related to housing because of the economic insecurity many experience due to discrimination and difficulty with employment opportunities [11].

Nationally, HIV viral suppression improved among clients served in the RWHAP between 2010 and 2017, and percentages of those receiving medical care through the program increased from 69.5% to 85.9% [9]. These improvements may be attributed to an increased focus on the HIV care continuum, utilizing data to drive outcomes, improvements in access to single dose HIV regimens, and changes in federal treatment guidelines that recommend HIV treatment at earlier timepoints regardless of CD4 counts and viral loads [12,13]. These changes, and the medical home model used by many HRSA RWHAP providers, have contributed to increased viral suppression and decreased racial, ethnic, and age disparities for clients served in the RWHAP [14,15]. For example, in 2010, the rate of viral suppression in youth ages 15–24 years old was 22.9 percentage points lower than the national average, whereas in 2017, this disparity gap decreased to 11.8 percentage points. However, for those same years, there was no decrease in this disparity gap for people experiencing unstable housing. The rate of viral suppression for people experiencing unstable housing remained at 14.7% below the national average in 2010 and 2017. Additionally, people identified as unstably housed have the lowest viral suppression rates (71.2%) when compared with other disproportionately affected groups such as youth ages 13–24 years old (74.1%) and transgender clients (80.9%) [9]. Although the RWHAP is able to fund temporary housing services, its primary intent is to fund essential healthcare services for people with HIV. Notwithstanding, when HRSA RWHAP leadership analyzed the client-level data, temporary or unstable housing status stood out as one of the most powerful indicators impacting lower HIV viral suppression rates across all populations. Given the fundamental role of stable housing in promoting positive health outcomes, it became apparent that focused efforts and partnerships were needed to address the healthcare needs of low-income people with HIV who access services in the program. HRSA leaders, therefore, engaged in a number of activities to make housing services more accessible to RWHAP clients, including aligning federal partnerships and leveraging resources, addressing policies to increase use of housing services, and funding initiatives to better understand how to address housing needs for people with HIV.

### Aligning federal partnerships and leveraging resources

HRSA partnered with the HUD, a federal agency that strengthens housing services by creating strong, sustainable, inclusive communities and quality affordable homes for all. In 2017, HRSA worked with HUD to jointly release a program statement to organizations and provider sites across both agencies. This emphasized and strongly encouraged their development of formal data-sharing processes and systems to increase coordination, integration, and services responsive to the housing and medical needs of people with HIV who are unstably or temporarily housed [16].

When HUD’s Housing Opportunities for Persons with AIDS (HOPWA) program changed its funding formula allocation in May 2017 to more equitably distribute funds, HRSA worked to understand the impact this would have on RWHAP providers. After identifying areas with significant increases or decreases in funding, HRSA and HUD jointly presented webinars at national conferences. These webinars outlined the potential impact of upcoming funding changes and provided technical assistance on how to leverage other funding sources for people with HIV who have housing needs.

### Addressing policies to increase use of housing services

In order to facilitate access to housing services, in 2016, HRSA modified and issued guidance on housing services to RWHAP providers. This guidance clarifies that clinical sites receiving RWHAP Part C funding, previously prohibited from providing housing services, could now support temporary housing services. Additionally, some reporting requirements have been reduced to increase accessibility to these services. The guidance also emphasizes their ability to provide emergency financial assistance to meet short-term emergency housing needs [17]. These changes demonstrate HRSA’s support of housing services and the agency’s ability to develop strategies to help find and provide people with HIV with housing.

### Funding initiatives to better understand how to address housing needs for people with HIV

HRSA is funding innovative housing models, interventions, and initiatives to share nationally with other provider sites. These activities gather best practices on (1) using multidisciplinary teams to support people with HIV experiencing homelessness and co-diagnosed with substance use disorders and/or mental disorders; (2) integrating housing and RWHAP data to ensure smoother transitions and that people with HIV do not fall out of the system of care as they are being linked to housing; and (3) addressing employment, financial stability, and housing through an integrated approach.

The models being tested in these studies have been developed by providers who serve people with HIV and are demonstrating some success. These include programs that engage unstably housed people with HIV by providing services that have a low threshold for easy accessibility, e.g., drop-in hours, and on-site access to vital housing and support services [18], merge RWHAP and housing data to better identify clients unstably housed and not retained in medical care [19], and integrate employment programs and housing subsidies into HIV care and treatment sites [20]. Many of these interventions and strategies show promising results, which could be replicated by other provider sites.

There is a growing understanding that stable housing is closely tied to health outcomes, particularly for low-income people in the US. The Centers for Medicare and Medicaid Services (CMS) provides healthcare coverage to millions of low-income people. Medicaid funds can not directly pay for housing but can pay for housing services (such as short-term rental payments or linkages to housing) [21]. Some states have started to use Medicaid funds for various preventive measures, including housing-related services that help individuals find and stay in housing. For example, California allows hospitals and social service organizations to collaboratively treat high-cost homeless people with HIV; Medicaid funds can be used for housing services, and local and state money can be applied directly to housing payments. North Carolina will launch pilot projects to use Medicaid funding for one-time security deposits and first month’s rent [22,23].

Collectively, these models and approaches are indicative of a clear understanding at the federal, state, and local levels of the importance of stable housing to positive clinical outcomes. Equally, models with promising outcomes that effectively help close health disparities associated with unstable housing among people with HIV may present opportunities for further research, propagation, and implementation by the RWHAP across provider sites.

### Looking to the future—Ending the HIV epidemic

As the US looks toward ending the HIV epidemic over the next 10 years, addressing the housing needs of people with HIV will play a critical part in meeting those bold goals. Data trends from the RWHAP have demonstrated that people with HIV who are unstably housed have the worst outcomes related to viral suppression regardless of race/ethnicity, gender, or poverty levels. Alongside the housing needs are the concomitant issues of mental health, substance use, and financial instability, which will also need to be prioritized and addressed. In order to affect significant gains, new partnerships with public and private stakeholders in the housing arena, listening to people with HIV who are homeless, and funding innovative strategies that address housing needs are needed. HRSA is well positioned to support those efforts and will play an important part in reaching those who are most in need of critical HIV and housing services including those not currently engaged in care.

### Disclaimer

The views expressed in this publication are solely the opinions of the authors and do not necessarily reflect the official policies of the US Department of Health and Human Services or the HRSA, nor does mention of the department or agency names imply endorsement by the US Government.

## Abbreviations

CMS: Centers for Medicare and Medicaid Services
HRSA: Health Resources and Services Administration
HUD: US Department of Housing and Urban Development
MSM: men who have sex with men
RWHAP: Ryan White HIV/AIDS Program
